# Thermal Acclimation of Foliar Carbon Metabolism in *Pinus taiwanensis* Along an Elevational Gradient

**DOI:** 10.3389/fpls.2021.778045

**Published:** 2022-01-10

**Authors:** Min Lyu, Mengke Sun, Josep Peñuelas, Jordi Sardans, Jun Sun, Xiaoping Chen, Quanlin Zhong, Dongliang Cheng

**Affiliations:** ^1^Key Laboratory of Humid Subtropical Eco-Geographical Processes, Ministry of Education, Fuzhou, China; ^2^Fujian Provincial Key Laboratory of Plant Ecophysiology, Fujian Normal University, Fuzhou, China; ^3^School of Urban and Rural Construction, Shaoyang University, Shaoyang, China; ^4^CSIC, Global Ecology Unit, CREAF-CSIC-UAB, Catalonia, Spain; ^5^CREAF, Cerdanyola del Vallès, Catalonia, Spain

**Keywords:** carbon metabolism, climate change, thermal acclimation, temperature sensitivity, *Pinus taiwanensis*

## Abstract

Climate change could negatively alter plant ecosystems if rising temperatures exceed optimal conditions for obtaining carbon. The acclimation of plants to higher temperatures could mitigate this effect, but the potential of subtropical forests to acclimate still requires elucidation. We used space-for-time substitution to determine the photosynthetic and respiratory-temperature response curves, optimal temperature of photosynthesis (*T*_opt_), photosynthetic rate at *T*_opt_, temperature sensitivity (*Q*_10_), and the rate of respiration at a standard temperature of 25°C (*R*_25_) for *Pinus taiwanensis* at five elevations (1200, 1400, 1600, 1800, and 2000 m) in two seasons (summer and winter) in the Wuyi Mountains in China. The response of photosynthesis in *P. taiwanensis* leaves to temperature at the five elevations followed parabolic curves, and the response of respiration to temperature increased with temperature. *T*_opt_ was higher in summer than winter at each elevation and decreased significantly with increasing elevation. *Q*_10_ decreased significantly with increasing elevation in summer but not winter. These results showed a strong thermal acclimation of foliar photosynthesis and respiration to current temperatures across elevations and seasons, and that *R*_25_ increased significantly with elevation and were higher in winter than summer at each elevation indicating that the global warming can decrease *R*_25._ These results strongly suggest that this thermal acclimation will likely occur in the coming decades under climate change, so the increase in respiration rates of *P. taiwanensis* in response to climatic warming may be smaller than predicted and thus may not increase atmospheric CO_2_ concentrations.

## Introduction

Climate change is becoming increasingly important as a global issue (Grosse et al., 2010; Sendall et al., 2015; Reich et al., 2016). Warming caused by climate change could negatively alter plant ecosystems if air temperatures exceed those optimal for obtaining carbon. Such changes may threaten temperature-sensitive species, causing local extinctions and migrations (Morgan-Kiss et al., 2006; Sendall et al., 2015). Photosynthesis and respiration are the two main physiological processes that link the biosphere and atmosphere in the global carbon cycle (King et al., 2006). Plants influence climate by exchanging energy, water, and other chemicals with the atmosphere (Lombardozzi et al., 2015). Future climatic warming throughout the ranges of species may lead to air and foliar temperatures that exceed current photosynthetic thermal optima, which could reduce photosynthetic capacity and carbon gain and thus negatively affect plant growth rates and survival (Sage and Kubien, 2007; Valladares et al., 2014). Understanding how these processes vary among different types of climate is a major goal for plant ecology (Wang et al., 2019).

Evidence suggests that temperature optima of species occur in parallel with latitudes and temperature isolines (Battaglia et al., 1996, Reich and Oleksyn, 2004, Sendall et al., 2015 and Kumarathunge et al., 2019). Several studies have reported that plants have higher thermal optima at lower than higher latitudes (Hill et al., 1988; Cunningham and Read, 2002), but others have found no evidence for a relationship between thermal optima and climatic distribution (Battaglia et al., 1996; Gunderson et al., 2000, 2010; Huang et al., 2019). The ability of species to adjust their photosynthetic optima to changes in temperature (i.e., acclimation) could limit reductions in gas-exchange rates (Berry and Bjorkman, 1980; Gunderson et al., 2010; Kattge and Knorr, 2010; Dusenge et al., 2020). Species growing near their colder, higher latitudinal limits may respond positively to warming, and such responses may be enhanced by gene flow (Davis and Shaw, 2001). Conversely, species growing near their warmer, lower latitudinal limits may have limited potential to respond to warming (Berry and Bjorkman, 1980; Tjoelker et al., 2008; Gunderson et al., 2010), and such responses may be delayed by the lack of gene flow from populations adapted to warmer temperatures, because individuals do not survive or are out-competed under the unfavorable conditions beyond their ranges (Davis and Shaw, 2001).

Plant respiration releases an annual flux of carbon dioxide (CO_2_) to the atmosphere, which will affect future climates (Slot et al., 2014a; Reich et al., 2016). A warming world may increase the respiratory release of CO_2_ because respiration responds positively to temperature and hence further atmospheric warming (Wang et al., 2020). Many studies have found that plants can dynamically adjust their respiration in response to temperature over the long term (weeks to years), even though increases in respiration always accelerate when subjected to a short-term (minutes to hours) increases in temperature, but the degree of acclimation is uncertain (Atkin and Tjoelker, 2003; Tjoelker et al., 2008; Slot and Kitajima, 2015). Observations of the acclimation of plants at different elevations and growing seasons are thus needed.

Elevational transects provide examples of plant trait variability along environmental gradients (Jian et al., 2009). This variability is partly related to the changes in air temperature with elevation (Xu et al., 2021). Therefore, elevation provides a method of the space-for-time substitution to predict trait variability in response to temperature and elevation gradients. *Pinus taiwanensis* is the dominant evergreen coniferous tree species that extends through a wide latitudinal and altitudinal range and the Wuyi Mountains is the most outstanding area for biodiversity conservation in southern China (Lyu et al., 2021). Its wide distribution provides a unique opportunity to study the physiological mechanisms responsible for tree thermal acclimation of subtropical forest. We assessed the capacity of *P. taiwanensis* in the Wuyi Mountains in China, to acclimate to warmer temperatures in summer and winter at five elevations along a gradient to advance our understanding of carbon metabolism in a changing climate. We measured the plasticity of thermal optima for photosynthesis and respiration rates. We assessed the magnitude of acclimation by comparing the photosynthetic and respiratory response curves of plants at different elevations and seasons. We tested the following hypotheses: (H1) *P. taiwanensi*s would exhibit a strong thermal acclimation of foliar photosynthesis and respiration to temperature along the elevational gradient, (H2) temperature acclimation would further modify the temperature optimum of *P. taiwanensis* in response to seasonal changes, and (H3) the increase in the respiration rates of *P. taiwanensis* acclimated to climatic warming would not increase atmospheric CO_2_ concentrations.

## Materials and Methods

### Site Description and Sampling

The experiment was conducted at the National Natural Reserve of the Wuyi Mountains (27°48.11′–28°00.35′N, 117°39.30′–117°55.47′E) in southeastern China. The reserve is in the humid warm subtropics and has a mean annual precipitation of 2583 mm and a mean annual temperature of 14.2°C. The average temperatures in July (summer) and December (winter) are 23.8 and 3.6°C, respectively. The air temperature decreases by 0.45 and 0.56°C with every 100-m increase in elevation in summer and winter, respectively. *P. taiwanensis* is distributed >1100 m a.s.l. We therefore established five sites along an elevational gradient: E1, E2, E3, E4, and E5 at 1200, 1400, 1600, 1800, and 2000 m, respectively. The soil N concentrations did not vary significantly with elevation. In contrast to soil N concentrations, the soil P concentrations increased significantly with elevation, from 0.19 ± 0.01 mg g^–1^ (mean ± standard error, SE) at E1 to 0.43 ± 0.02 mg g^–1^ (mean ± standard error, SE) at E5 (Lyu et al., 2021).

We selected the mature individuals about 30–50 years old. Furthermore, to remove the biological influence of tree age on decreasing growth at higher elevation, we selected current-year branch (without apparent leaf area loss) and collected fully mature needles to measure the carbon flux. We established three 20 × 20 m plots at each elevation. Three trees were selected in each plot. Three branches with tips at the outer edge of the crown were randomly selected for each tree in summer (July) and winter (December) in 2017. A total of 90 branches (five elevations × three plots × three trees × two seasons) were selected.

### Measurement of Foliar Gas Exchange

Fully mature needles were collected from each branch selected (without apparent loss of foliar area). Photosynthetic and respiration rates were measured using an LI-6800 portable photosynthesis system (LI-COR, Lincoln, United States), and temperature response curves were developed based on measurements at 17, 22, 27, 32, and 37°C in summer and 5, 10, 15, 20, 25 and 30°C in winter. The light level in the leaf chamber was maintained at 2,000 μmol m^–2^ s^–1^, air flow was set at 300–500 μmol s^–1^, and CO_2_ concentration was set at 400 μmol mol^–1^. Net assimilation rate was measured from 09:00 to 12:00. The rate of dark respiration (*R*_d_) was measured using needles shaded with a black cloth for 1 h. *R*_d_ under these conditions is stable in detached leaves for several hours or longer (Reich et al., 2016). Measurements were made in July (summer) and December (winter) from 1200 to 2000 m. All plants were measured in one elevation over 3–5 days.

*Q*_10_ of the temperature-response function for each leaf, and the respiration rate at a standard measurement temperature of 25°C (*R*_25_), were calculated using the temperature-response equations proposed by Slot et al. (2013, 2014b) and Reich et al. (2016):


(1) ln(R)=a+bT

where, a and b are, respectively the intercept and the slope of the response curve. *Q*_10_ values were calculated from the slope of these equations as:


(2) Q10=e10 b

*R*_25_ was calculated for each of the 5–7 set cuvette temperatures of each leaf as:


(3) $$R_{25}=e^{a\text{​ }+\text{ bT }{\text{+cT}}^{\text{2}}}$$

where, *R*_25_ is dark respiration measured at a leaf temperature of 25°C, and *a*, *b*, and *c* are coefficients that describe the response of the natural log of respiration to temperature.

### Fitting Response Curves of Photosynthetic Temperature

The photosynthetic thermal optimum for each leaf measured was estimated using nonlinear regression of the data for photosynthetic thermal response:


(4) $$A_{\left(\text{T}\right)}=A_{\text{opt}}-b{\left(T-T_{\text{opt}}\right)}^{2}$$

where, *A*_(T)_ is the measured net rate of CO_2_ assimilation (μmol m^–2^ s^–1^) at foliar temperature *T*, *b* is a parameter for the spread of the parabola (Battaglia et al., 1996), *T*_opt_ is the optimal temperature for photosynthesis, and *A*_opt_ is the rate of photosynthesis at *T*_opt_.

### Data Analysis

The foliar values were averaged. Mixed-effects analyses of variance (ANOVAs) were used to compare *T*_opt_, *A*_opt_, and parameter *b*. The influence of elevation on *T*_opt_, *A*_opt_, *R*_25_, and *Q*_10_ was analyzed using LSD tests and multivariate analyses of variance (multiple-comparisons ANOVAs) using the *agricolae* package in R version 3.4.4. These variables were assessed using IBM SPSS Statistics V.22.0 (International Business Machines Corporation, Armonk, United States). The level of significance for testing slope heterogeneity was *P* < 0.05 (i.e., slope heterogeneity was rejected if *P* > 0.05). An LSD test and a *t*-test were used to analyze the variance. The data for elevation and season did not differ significantly when α was examined to identify common scaling exponents using the standardized major-axis package in R.

The allometric relationships between *T*_opt_ and *A*_opt_ were described after log 10-transformation. A scaling approach consisted of *y* = βx^α^ (Eq. 4), where y and x are *T*_opt_ and *A*_op_, respectively, β is the normalization constant (intercept), and α is the scaling exponent (slope). The equation describes an isometric relationship when α = 1 and an allometric relationship when α ≠ 1. Eq. 4 was log_10_-transformed to log_10_ *y* = log10 (β) + α log_10_ x and then fitted using model II standardized major-axis regression of the “smatr” package (Warton et al., 2006). A common scaling exponent was calculated when the scaling exponents did not differ significantly (*P* > 0.05) among the groups.

## Results

The temperature response curves of photosynthesis for *P. taiwanensis* leaves at different elevations followed parabolic curves for both summer and winter. The photosynthetic rate increased with temperature and then decreased when the temperature exceeded the optimum.

**FIGURE 1:**
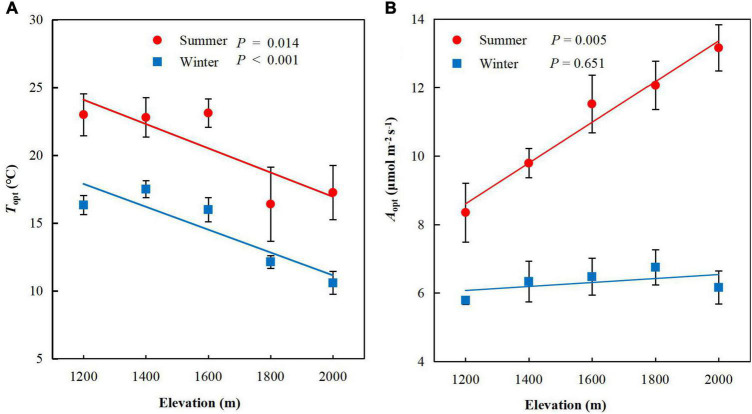
Photosynthetic thermal optimum and rate of CO_2_ assimilation of *Pinus taiwanensis* sampled at five elevations in the Wuyi Mountains. **(A)** Mean foliar photosynthetic thermal optimum (*T*_opt_), and **(B)** rate of CO_2_ assimilation at *T*_opt_ (*A*_opt_).

Elevation significantly negatively affected *T*_opt_ in summer (*P* = 0.014, Figure 1A) and winter (*P* < 0.001). In contrast to *T*_opt_, *A*_opt_ increased significantly with elevation in summer (*P* = 0.005, Figure 1B) but not winter (*P* = 0.651). *T*_opt_ decreased by 1.62°C for every 1°C decrease in growth temperature across of *P. taiwanensis* five elevations in the Wuyi Mountains, accompanied by increases in 1.34 μmol m^–2^ s^–1^ of *A*_opt_ (Table 1).

**TABLE 1:** Mean (± standard error) foliar photosynthetic thermal optimum (*T*_opt_), rate of CO_2_ assimilation at *T*_opt_ (*A*_opt_) and the rate of respiration at a standard temperature of 25°C (*R*_25_) for *Pinus taiwanensis* sampled in growth temperatures (*T*_growth_) at five elevations in the Wuyi Mountains.

Elevation (m)	*T*_growth_ (°C)	*T*_opt_ (°C)	*A*_opt_ (μmol m^–2^ s^–1^)	*R*_25_ (μmol m^–2^ s^–1^)
1200	23.26	23.00 ± 1.54	8.35 ± 0.86	1.1 ± 0.04
1400	22.20	22.79 ± 1.45	9.79 ± 0.43	0.97 ± 0.03
1600	21.50	23.12 ± 1.05	11.52 ± 0.84	1.25 ± 0.07
1800	20.60	16.40 ± 2.73	12.07 ± 0.71	2.1 ± 0.11
2000	19.40	17.26 ± 2.00	13.16 ± 0.67	1.92 ± 0.10

**FIGURE 2:**
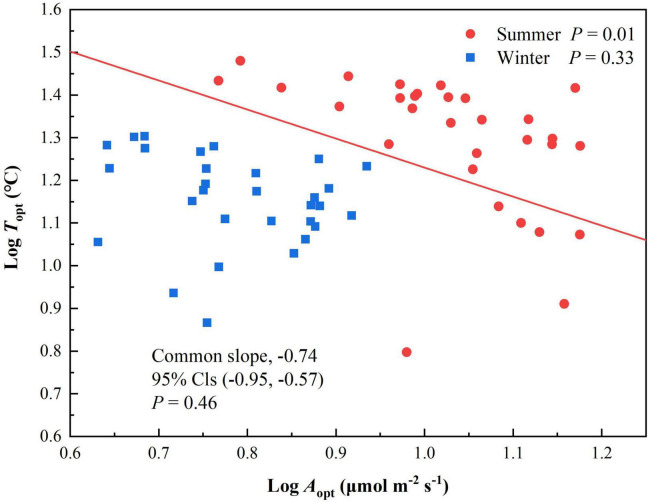
Scaling relationships of *T*_opt_ and *A*_opt_ for *Pinus taiwanensis* sampled at five elevations in the Wuyi Mountains. Lines are significant standardized major-axis regressions (*P* < 0.05).

*T*_opt_ and *A*_opt_ at each elevation were higher in summer than winter. *A*_opt_ was significantly correlated with *T*_opt_ in summer (*P* = 0.01, Table 2) but not winter (*P* = 0.33. The scaling slopes of *T*_opt_ and *A*_opt_ in summer and winter did not differ significantly across the five elevations and had a common slope of −0.74 (95% confidence intervals (CIs) = −0.95 and −0.57, *P* = 0.46, Figure 2). The normalization constants for *T*_opt_ vs *A*_opt_, however, varied significantly (*P* < 0.001), ranging from 1.73 (95% CIs = 1.37 and 2.09) for winter to 1.91 (95% CIs = 1.60 to 2.21) for summer.

**TABLE 2:** Summary of regression slopes and y-intercepts (α and log β, respectively) for the relationship between foliar *T*_opt_ and *A*_opt_ for *Pinus taiwanensis* sampled at five elevations in the Wuyi Mountains.

Log y vs log x	α (95% CIs)	Log β (95% CIs)	*r* ^2^	*P*	*P*_–1.0_	*n*
Summer	−0.68 (−0.95, −0.48)	1.91 (1.60, 2.11)	0.21	0.01	0.02	30
Winter	−0.82 (−1.19, −0.56)	1.73 (1.37, 2.09)	0.03	0.33	0.28	30

*P_–1.0_ indicates a significant difference between the slope and a slope of 1.0 at P < 0.05. 95% CIs, 95% confidence intervals.*

**FIGURE 3:**
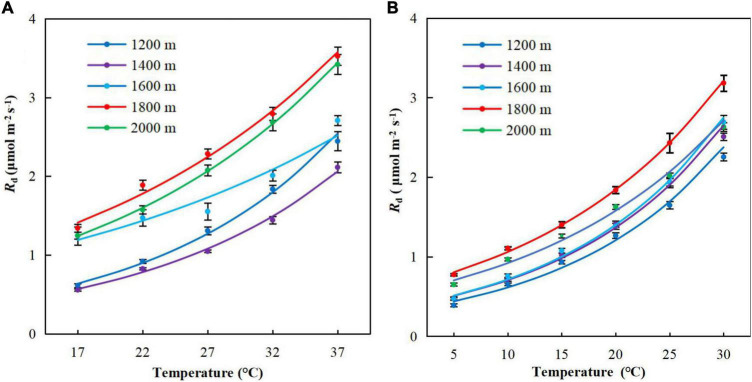
Temperature response curves of respiration for *Pinus taiwanensis* sampled at five elevations in the Wuyi Mountains. **(A)** Summer and **(B)** winter. Error bars indicate standard errors.

The respiratory temperature response curves displayed a characteristic sustained increase with temperature (Figure 3). The respiration rate increased slowly from 5 to 20°C and then increased rapidly when the temperature in the leaf chamber exceeded 25°C. The respiration rates were higher at high elevations (E3–5) than low elevations (E1–3).

**FIGURE 4:**
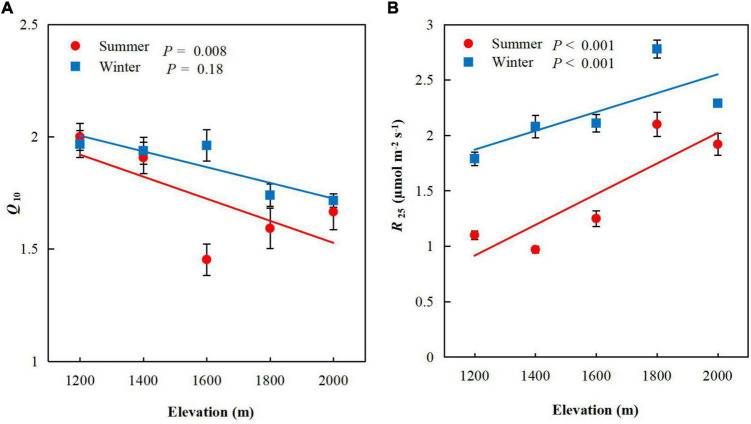
Mean foliar **(A)** *Q*_10_ and **(B)** *R*_25_ for *Pinus taiwanensis* sampled at five elevations in the Wuyi Mountains. Error bars indicate standard errors.

*Q*_10_ decreased significantly as elevation increased (*P* < 0.001, Figure 4A) in summer (*P* = 0.008), but not winter (*P* = 0.18). The mean values of *Q*_10_ was higher in winter (mean 1.86, range 1.72–1.97) than summer (mean 1.72, range 1.45–2.00), but did not differ significantly between seasons (Table 3). *R*_25_ increased significantly with elevation, from 1.1 ± 0.04 μmol m^–2^ s^–1^ (mean ± standard error, SE) at E1 to 1.92 ± 0.10 μmol m^–2^ s^–1^ (mean ± SE) at E5 in summer (*P <* 0.001, Figure 4B) and from 1.79 ± 0.06 μmol m^–2^ s^–1^ (mean ± SE) at E1 to 2.29 ± 0.03 mg g^–1^ (mean ± SE) at E5 in winter (*P* < 0.001). We chose to use *R*_25_ because it is widely reported in the literature and used for comparison of respiration rates of plants from different biomes, and 25°C is above the average temperature of the sampling sites, which had a mean annual temperature of 14.2°C (Slot et al., 2014b; Reich et al., 2016; Way et al., 2019; Lyu et al., 2021).

**TABLE 3:** Results of a two-way ANOVA of *Q*_10_ for *Pinus taiwanensis* leaves for season, elevation.

*Q*_10_	*F*	*P*
Season	1.46	0.29
Elevation	1.59	0.24

## Discussion

### Potential of Photosynthesis to Acclimate to Temperature

The relationship between temperature and photosynthetic rate can generally be described with a parabolic curve, in which the rate increases before reaching the optimal temperature and then decreases (Battaglia et al., 1996; Thuiller et al., 2005; Walker et al., 2006). Our findings were consistent with this relationship; *P. taiwanensis* had a higher photosynthetic rate under *T*_opt_ conditions. The ranges of *T*_opt_ in our study were 19.25–23.6 and 10.68–17.63°C in summer and winter, respectively, and the average temperatures in summer and winter at our experimental site were 23.8 and 3.6°C, respectively (Lyu et al., 2021). These conditions indicate that rising global temperatures (of 1.1–6.4°C by 2100) (Intergovernmental Panel on Climate Change [IPCC], 2013) could increase the photosynthetic rate in *P. taiwanensis*, especially in winter. Optimal thermal acclimation could ensure the maximum absorption of CO_2_ by plants and reduce CO_2_ concentration in the atmosphere (Sendall et al., 2015).

*T*_opt_ was higher in summer than winter along the gradient and decreased significantly as elevation increased (Figure 1A). This suggests that temperature acclimation would further modify the temperature optimum in response to seasonal changes of *P. taiwanensi*s. Elevation significantly affected *T*_opt_. Species growing near their warmer, lower elevational limits, where boundaries are partly determined by thermal limitations (Berry and Bjorkman, 1980; Tjoelker et al., 1998, 2008; Davis and Shaw, 2001), Gunderson et al., 2010) or increased levels of competition, may be constrained in their potential to acclimate to warming (Reich et al., 2015). In contrast, species growing near their colder, higher elevational limits may respond more strongly to environmental change. This finding provides further evidence that species have capacities to acclimate relative to changing temperatures. Under a future warming scenario *P. taiwanensis* will move from lower to higher altitudes, probably ceding its dominance at lower altitudes but expanding to higher altitudes such is being observed in several sites along the world for other forest species (Peñuelas and Boada, 2003; Peñuelas et al., 2007).

*A*_opt_ in winter did not differ significantly along the elevational gradient, and *A*_opt_ was correlated with *T*_opt_ in summer, but not in winter. The scaling slopes of *T*_opt_ and *A*_opt_ did not differ significantly between summer and winter, but the normalization constants varied significantly, perhaps because low temperatures limit photosynthesis in alpine species in winter (Sendall et al., 2015; Lyu et al., 2021). Many studies (Smith and Dukes, 2013; Sendall et al., 2015; Smith et al., 2015) have reported that the photosynthetic rate of leaves are affected by low temperatures. Photosynthesis can also be strongly influenced by environmental factors such as light and water (Körner, 1998; Valladares et al., 2014), which may also account for the lack of significant differences in winter maximum photosynthetic rate among the elevations.

### Sensitivity of *Pinus taiwanensis* to Temperature

Respiration rates is generally assumed to double with 10°C temperature increase; that is, it has a *Q*_10_ (the proportional increase in respiration rates with 10°C warming) of 2.0 (Slot et al., 2014a). Many studies (Stockfors and Linder, 1998; Atkin and Tjoelker, 2003; Tjoelker et al., 2008) have found that *Q*_10_ decreased with increasing temperature, inconsistent with our study. *Q*_10_ in our study, however, was decreased with decreasing temperature in summer. The mean *Q*_10_ value was higher in winter than summer.

The respiration rate of plant leaves is extremely sensitive to changes in temperature over short timescales (several minutes); Ecosystems and plant environments, however, are controlled and regulated by the long-term threshold of the temperature of the environment but also may be affected by their own plant growth and development, including changes in foliar morphology, matrix, and nutrient status.

*Q*_10_ decreased significantly with increasing elevation in summer, indicating that the respiratory sensitivity of *P. taiwanensis* leaves decreased significantly with decreasing temperature. This finding is consistent with a previous study on the foliar NSC concentrations where the rate decreased significantly as elevation increased (Lyu et al., 2021). *P. taiwanensis* is insensitive to low temperatures, which is beneficial for increasing the storage of carbohydrates, thus providing effective resource use for developing mechanisms to acclimate to high levels of stress.

As plants become less sensitive to environmental changes over time (i.e., they acclimate), the initial response can represent the instantaneous characteristics of plants. Our results indicated that *P. taiwanensis* could acclimate to environments with low temperatures by reducing its instantaneous sensitivity to temperature. It could thereby obtain the minimum amount of carbon necessary for survival, which could be an important strategy of carbon metabolism for survival at alpine treelines.

### Do Increased Respiration Rates Increase Atmospheric Carbon Dioxide Concentrations?

Climatic warming may increase plant respiration, increasing the release of CO_2_ from terrestrial ecosystems and further increasing atmospheric warming. The respiratory response to temperature in our study increased with temperature at the five elevations in both summer and winter. *R*_25_, however, was highest at E5 (2,000 m) and decreased toward E1 (1,200 m), with 42.7 and 21.83% decreases between E5 and E1 in summer and winter, respectively, indicating that the rate of respiration decreased with increasing temperature. Stress due to high temperatures can lead to respiratory acclimation and thereby reduce respiration (Reich et al., 2016). Plant respiration always increases in response to a short-term increase in temperature, but responses can vary over the long term (Teskey and Will, 1999; Smith and Dukes, 2013; Reich et al., 2016). A plant that has experienced warmer temperatures will typically have a rate of respiration at a given temperature lower than a plant that has experienced cooler temperatures (Slot et al., 2014a; Wang et al., 2020).

As plant respiration responds positively to temperature, a warming world may result in additional respiratory CO_2_ release, and hence further atmospheric warming (Atkin et al., 2006; Reich et al., 2016). In our study, *R*_25_ increased significantly with elevation and was higher in winter than summer at each elevation indicating that the warming can decrease of respiration.

Furthermore, Lyu et al. (2021) found that the respiration rates for *P*. *taiwanensis* increased with elevation in summer. It indicate that respiration rates of *P. taiwanensis* can acclimate to altered temperatures and weakening the positive feedback of plant respiration to rising global air temperature. Thus the increase in respiration rates of *P. taiwanensis* in response to climatic warming may be smaller than predicted and thus may not increase atmospheric CO_2_ concentrations. The populations acclimated to lower altitude thus to high temperatures have lower *R*_d_ and thus a clear acclimation capacity to decrease *R*_d_ when temperatures rise permanently and the population has had time enough to acclimate by reducin*g R*_d_. Lombardozzi et al. (2015) and Sendall et al. (2015) suggested that foliar respiratory acclimation globally may have a larger ameliorating impact than expected on CO_2_ losses with rising temperatures under climate change. Such amelioration would be even larger if stems and roots acclimated similarly to leaves, which require further research (Reich et al., 2016).

## Conclusion

Foliar carbon metabolism in *P. taiwanensis* strongly acclimated to temperature across elevations and seasons. These findings indicated that *P. taiwanensis* could adapt to low temperatures by reducing its sensitivity to temperature and obtaining the minimum amount of carbon necessary for survival, which is an important strategy of carbon metabolism and has likely allowed this species to be able to grow in high montane forests. Rising global temperatures will probably increase the photosynthetic rate of *P. taiwanen*sis, but the increase in the respiration rate in response to climatic warming may be smaller than predicted and thus may not increase atmospheric CO_2_ concentrations. Our results provide field evidence for the adaptation of plant carbon metabolism in a changing climate. This information could be used in models of climate change and contributes to our understanding of the consequences of acclimation on carbon cycling.

## Data Availability Statement

The raw data supporting the conclusions of this article will be made available by the authors, without undue reservation.

## Author Contributions

ML, MS, QZ, and DC conceived and designed the experiments. XC and JSu performed the experiments. XC, ML, MS, and DC analyzed the data. ML, MS, QZ, and DC wrote the manuscript. JP and JSa performed the research and revised the manuscript. All authors approved the final version of the manuscript.

## Conflict of Interest

The authors declare that the research was conducted in the absence of any commercial or financial relationships that could be construed as a potential conflict of interest.

## Publisher’s Note

All claims expressed in this article are solely those of the authors and do not necessarily represent those of their affiliated organizations, or those of the publisher, the editors and the reviewers. Any product that may be evaluated in this article, or claim that may be made by its manufacturer, is not guaranteed or endorsed by the publisher.
