# Supplementary material for: Thermal Acclimation of Foliar Carbon Metabolism in Pinus taiwanensis Along an Elevational Gradient
Source: Front Plant Sci. 2022 Jan 10;12:778045. doi: 10.3389/fpls.2021.778045 (PMC8784779; doi:10.3389/fpls.2021.778045)
Supplement: Supplementary file 1 [file Data_Sheet_1.PDF]

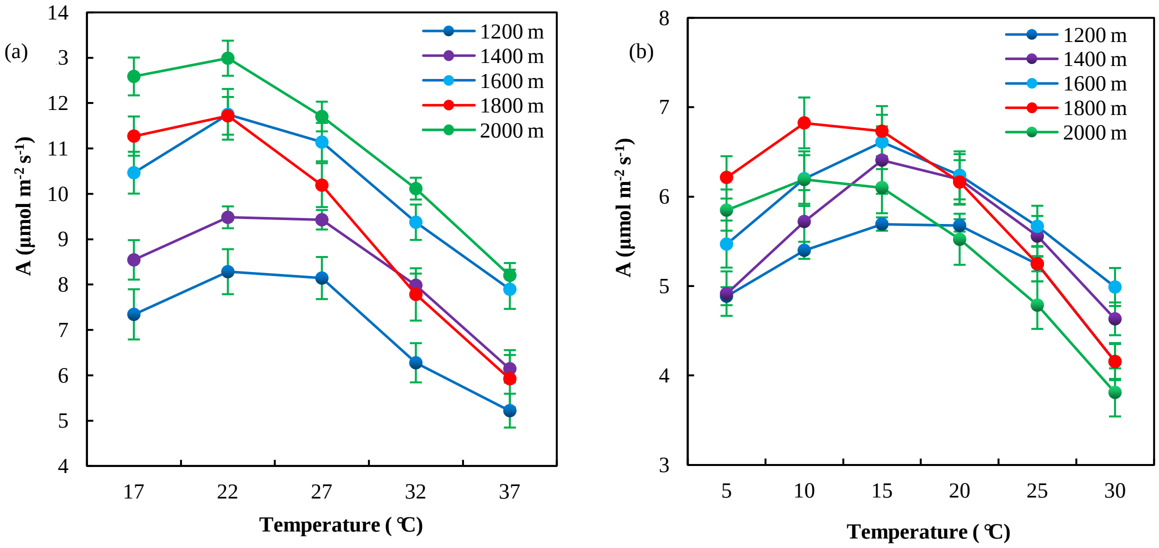

Figure S1 Response curves of mean photosynthetic temperature of *Pinus taiwanensis* sampled at five elevations in the Wuyi Mountains. **(a)** Summer and **(b)** winter. Error bars indicate standard errors.
